# The transcription factor Prox1 is essential for satellite cell differentiation and muscle fibre-type regulation

**DOI:** 10.1038/ncomms13124

**Published:** 2016-10-12

**Authors:** Riikka Kivelä, Ida Salmela, Yen Hoang Nguyen, Tatiana V. Petrova, Heikki A. Koistinen, Zoltan Wiener, Kari Alitalo

**Affiliations:** 1 Wihuri Research Institute, Biomedicum Helsinki, Haartmaninkatu 8, Helsinki 00290, Finland; 2 Translational Cancer Biology Program, Faculty of Medicine, University of Helsinki, P.O. Box 63, Helsinki 00014, Finland; 3 Minerva Foundation Institute for Medical Research, Biomedicum Helsinki 2U, Tukholmankatu 8, Helsinki 00290, Finland; 4 Department of Medicine and Abdominal Center: Endocrinology, University of Helsinki and Helsinki University Central Hospital, Haartmaninkatu 4, P.O. Box 340, Helsinki 00029, Finland; 5 Department of Fundamental Oncology, Centre Hospitalier Universitaire Vaudois (CHUV) and University of Lausanne (UNIL), and Division of Experimental Pathology, Institute of Pathology, CHUV, CH-1066 Epalinges, Switzerland

## Abstract

The remarkable adaptive and regenerative capacity of skeletal muscle is regulated by several transcription factors and pathways. Here we show that the transcription factor Prox1 is an important regulator of myoblast differentiation and of slow muscle fibre type. In both rodent and human skeletal muscles Prox1 is specifically expressed in slow muscle fibres and in muscle stem cells called satellite cells. Prox1 activates the NFAT signalling pathway and is necessary and sufficient for the maintenance of the gene program of slow muscle fibre type. Using lineage-tracing we show that Prox1-positive satellite cells differentiate into muscle fibres. Furthermore, we provide evidence that Prox1 is a critical transcription factor for the differentiation of myoblasts via bi-directional crosstalk with Notch1. These results identify Prox1 as an essential transcription factor that regulates skeletal muscle phenotype and myoblast differentiation by interacting with the NFAT and Notch pathways.

## Abstract (web summary)

Skeletal muscle has remarkable adaptive and regenerative capacity. Here the authors show that the transcription factor Prox1 is necessary for maintenance of slow muscle fibre types via activation of NFAT signalling, and for myoblast differentiation via cross-talk with the Notch signalling pathway.

## Graphical abstract

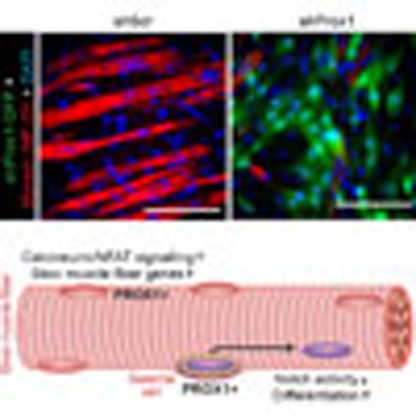

Adult skeletal muscles regenerate after damage due to the proliferation and differentiation of muscle resident stem cells called satellite cells (SC). In healthy resting muscle these cells are in a quiescent state, which is characterized by a reversible mitotic arrest and slow metabolic activity indicative of many stem cell populations^1^. In response to muscle injury, SCs can be activated, resulting in proliferation, differentiation and self-renewal^2^. Members of the MyoD family of myogenic regulatory factors (MyoD, Myogenic factor 5 (*Myf5*), Myogenic factor 6/Mrf4 and myogenin) control the formation of skeletal muscle and muscle regeneration in the adult^2^. The paired box factors Pax3 and Pax7 are important upstream regulators of the myogenic regulatory factors in SCs and the differentiation of committed myoblasts require the downregulation of Pax7 (refs 3, 4). However, the molecular mechanisms underlying SC activation and differentiation are not completely understood.

Skeletal muscle fibres can be classified by their contraction speed and their aerobic (oxidative) versus anaerobic (glycolytic) production of ATP^5^. Slow type I fibres express myosin heavy chain I (*Myh7,* MyHC I), use aerobic metabolism, are rich in mitochondria and myoglobin, and have good endurance. In contrast, type IIB fast twitch fibres, which express *Myh4*/MyHC IIB, are primarily anaerobic and glycolytic, contain many glycogen granules, and contract quickly for short-term activity. In addition, the oxidative type IIA (*Myh2*/MyHC IIA) and intermediate type IIX (*Myh1*/MyHC IIX) fast twitch fibres have intermediate contraction properties compared with type I and IIB fibres.

The prospero-related homeobox 1 (Prox1) transcription factor, especially its homeo-prospero domain, is highly conserved among vertebrates^6^,^7^,^8^. Prox1 is essential for the development of several organs; Prox1-deficient mice die before birth due to multiple developmental defects^9^. A role for Prox1 in the development of lymphatic vasculature, liver, pancreas, heart and lens has been shown in previous studies^9^,^10^,^11^,^12^,^13^. Some studies have suggested a role for the Prox1 in skeletal muscle development and fibre type specification^14^,^15^,^16^. In zebrafish, the U-boot (*Ubo*) gene, which regulates slow versus fast fibre differentiation pathways, activates Prox1 in slow myoblasts. This is crucial for the terminal stages of fibre development and for their proper organization in slow fibres^14^. Recently, Petchey *et al*.^16^ demonstrated in mice that Prox1 is required for the repression of fast troponin T3, troponin I2, and myosin light chain 1 in both cardiac and skeletal muscle, indicating conservation of Prox1 function in vertebrates^15^.

Here we assess the role of Prox1 in the regulation of skeletal muscle phenotype and function. We show that Prox1 is expressed in rodent and human slow muscle fibres and in SC. In SC, Prox1 expression is induced on differentiation, and Prox1 silencing results in Notch1 activation and inhibition of myoblast differentiation. Interestingly, Prox1 is also necessary and sufficient for the slow fibre type gene program via regulation of NFAT signalling.

## Results

### Prox1 is expressed in slow muscle fibres and SC

**Figure 1:**
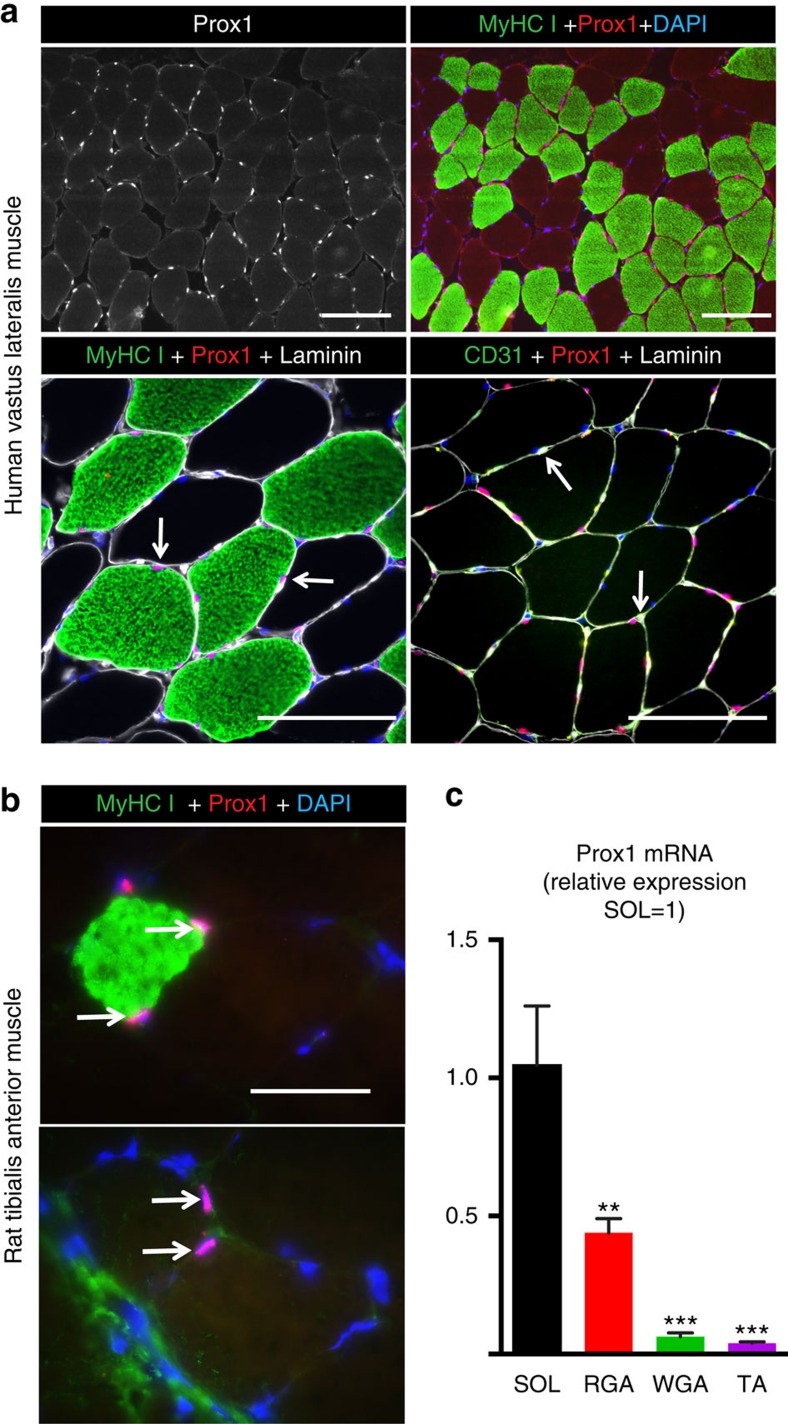
Prox1 expression in slow muscle fibres in mammalian skeletal muscle. (**a**) Prox1 expression in the nuclei (magenta) of slow muscle fibres stained for MyHC I (green) in human vastus lateralis muscle. Co-staining with laminin indicates that the Prox1 positive nuclei are under the basal lamina of the muscle fibres (arrows). Note that Prox1 staining does not co-localize with endothelial cells (CD31, arrows). (**b**) Prox1 positive nuclei (magenta) in MyHC I (green) positive slow muscle fibres in rat tibialis anterior muscle. Few Prox1 positive nuclei were found also in the proximity of fast fibres. (**c**) Prox1 mRNA expression in various rat skeletal muscles. Note the highest expression in the slow soleus (SOL) muscle. RGA, red gastrocnemius; TA, tibialis anterior; WGA, white gastrocnemius. Data is presented as mean±s.e.m., *n*=4, one-way analysis of variance with Tukey's *post hoc* test, **P<0.01, ***P<0.001 compared with soleus. Scale bars, 50 μm.

Prox1 expression in skeletal muscle has been reported in zebrafish and mice^14^,^15^,^16^. Our results show that human skeletal muscle fibres express Prox1 and, interestingly, we observed nuclear Prox1 immunostaining only in the nuclei of muscle fibres that express the slow MyHC I in all mouse, rat and human skeletal muscles studied (Fig. 1a,b). The Prox1-positive nuclei were located under the basal lamina, indicating that they were muscle fibre nuclei. Furthermore, Prox1 was not expressed in blood vascular endothelial cells. We compared Prox1 expression in different rat muscles, and observed the highest Prox1 mRNA levels in the soleus muscle (SOL), which predominantly consists of type I slow fibres, followed by the red part of the gastrocnemius muscle (RGA) enriched for type IIA fibres (Fig. 1c). In contrast, Prox1 expression was only marginal in the tibialis anterior (TA) and the white part of the gastrocnemius muscle (WGA), both containing mainly fast IIB fibres. Mouse and rat skeletal muscles contain fewer slow fibres than most human muscles and, in accordance with this, fewer Prox1-positive nuclei were observed in rodent muscles than in human muscles (Fig. 1).

**Figure 2:**
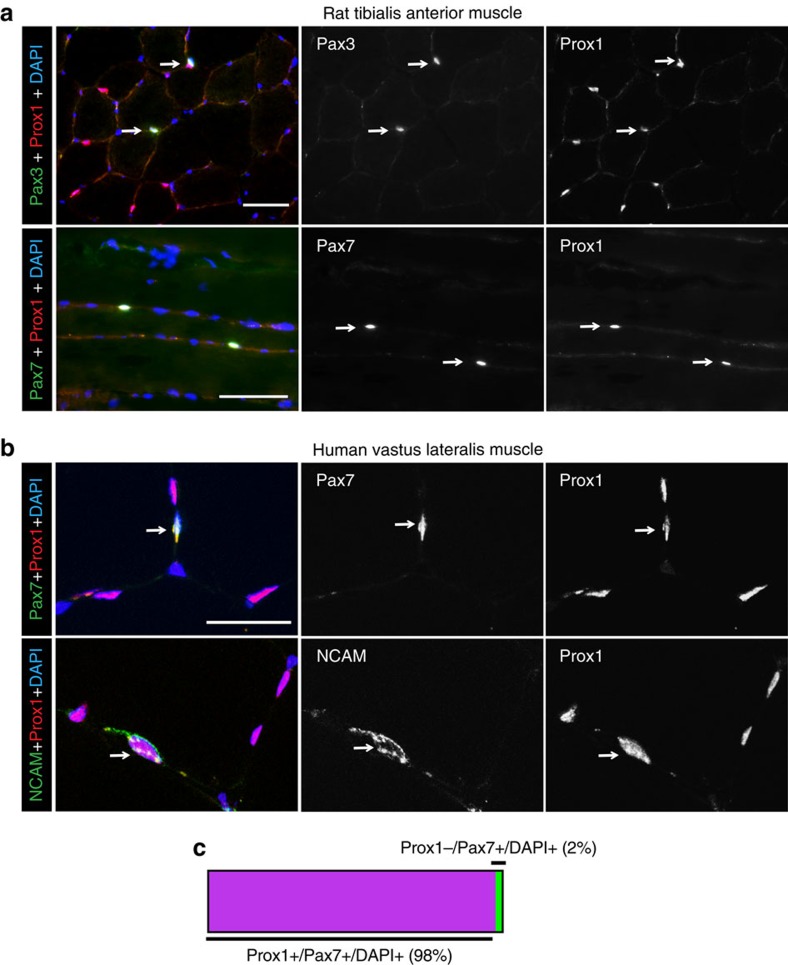
Prox1 is expressed in rodent and human satellite cells. (**a**) Prox1 colocalization with Pax3 and Pax7 positive satellite cells (arrows) in quiescent rat tibialis anterior muscle. (**b**) Prox1 colocalization with Pax7 and NCAM positive satellite cells (arrows) in human vastus lateralis muscle. (**c**) A bar graph showing the percentage of Prox1+ cells amongst Pax7+/Dapi+ cells in human vastus lateralis muscle. A total of 83 cells were analysed from five subjects. Scale bars, 50 μm.

In addition, we observed mononuclear MyHC I-negative cells expressing Prox1 in the skeletal muscle. Staining of rat TA muscle sections for the SC markers Pax3 and Pax7 with Prox1 indicated that these cells have SC identity (Fig. 2a), raising the possibility that Prox1 has a role in the regulation of muscle stem cells as well. We further confirmed our finding in human skeletal muscles, where Pax7 and NCAM (CD56) positive SCs expressed Prox1 (Fig. 2b), with 98% of Pax7+ nuclei being Prox1 positive (81 out of 83 cells) (Fig. 2c).

### Prox1 expression is necessary for the slow muscle phenotype

**Figure 3:**
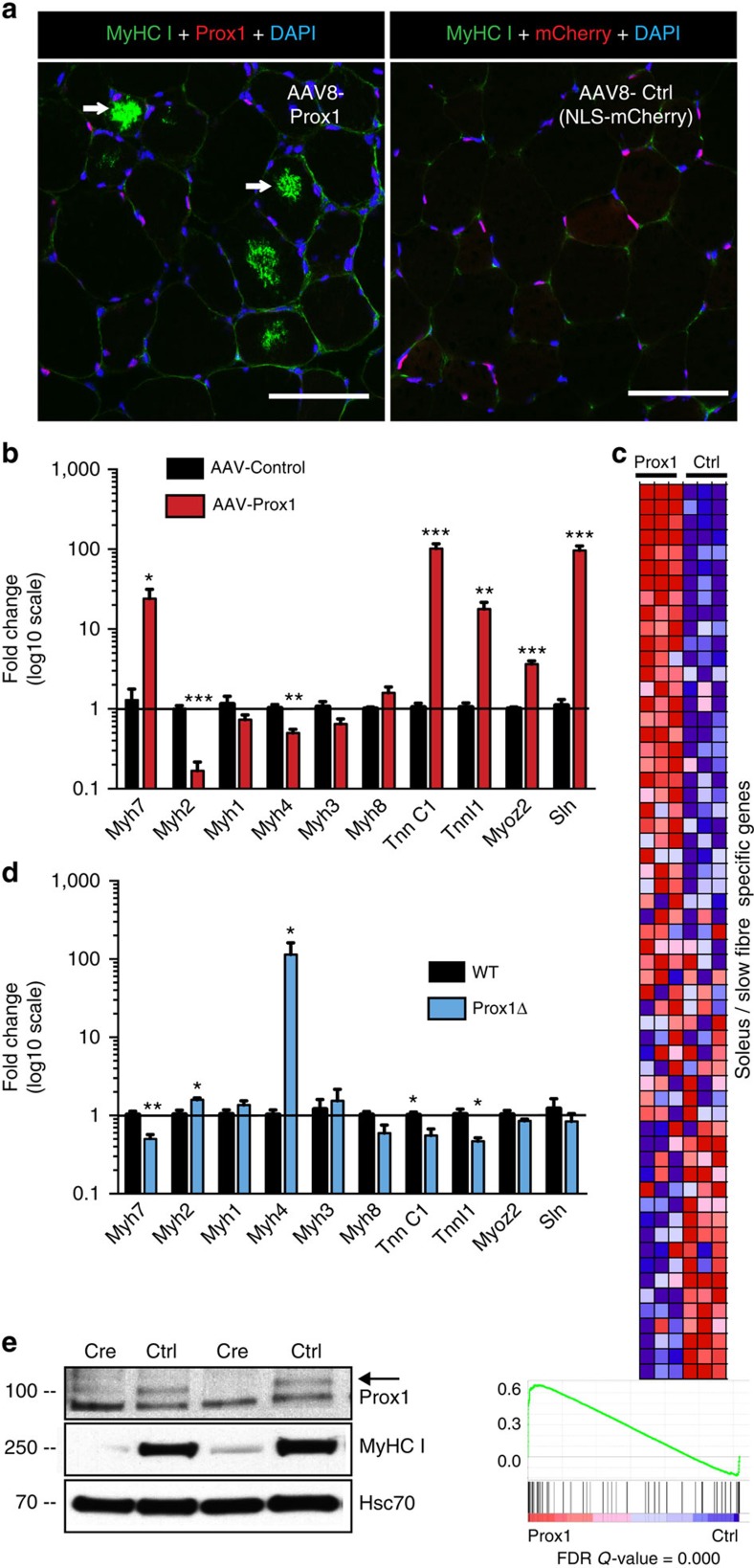
Prox1 regulates the slow muscle fibre gene program in skeletal muscle. (**a**) MyHC I (Myh7) expression in muscle fibres (arrows) in AAV8-Prox1 and AAV8-Ctrl transduced TA muscle. (**b**) Expression of myosin heavy chain and calcium-signalling genes in AAV8-Prox1 and AAV8-Ctrl transduced TA muscle. Note that the expression of slow fibre -specific genes are upregulated and fast MyHC genes downregulated in Prox1 overexpressing muscles. (**c**) Heat map from GSEA analysis showing that Prox1 overexpression in TA muscle leads to expression of many soleus specific myofibrillar genes (red colour indicates positive and blue negative enrichment). (**d**) Effect of Prox1 deletion on soleus muscle gene expression (HSA-Cre^ERT2^;Prox1^fl/fl^ mice). Note the increased expression of fast MyHCs Myh2 and Myh4 RNAs, whereas slow troponin isoform RNAs and Myh7 RNA are decreased. (**e**) Deletion of Prox1 in slow soleus muscle with AAV-Cre leads to marked downregulation of MyHC I protein. Data is presented as mean±s.e.m., *n*=5+5 in **a**,**b**, *n*=3+3 in **c**, *n*=4+4 in **d**,**e**. Student's two-tailed unpaired *t*-test, *P<0.05, **P<0.01, ***P<0.001. Both overexpression and deletion experiments were conducted two times on independent mouse cohorts. WT, wild-type. Scale bars, 50 μm.

Since Prox1 was abundantly expressed in slow muscle fibres, we next assessed the function and downstream signalling of Prox1 in skeletal muscles. Adeno-associated viral vector (AAV8) encoding Prox1 was injected into mouse TA muscle, which is composed of fast muscle fibres with very low endogenous Prox1 expression. The contralateral muscle was injected with AAV encoding mCherry reporter protein equipped with a nuclear localization signal. Although the overall muscle structure was not affected by Prox1 overexpression, after four weeks we observed occasional cells expressing MyHC I (Fig. 3a), which were not found in the control TA muscles (MyHC I staining in 6.6 % of all fibres and 25.4 % of Prox1 overexpressing fibres).

Prox1 transduction to fast skeletal muscle resulted in the activation of the slow muscle fibre specific gene program involving contractile elements and calcium regulation (Myh7, TnnC1, TnnCI1, Myoz2, Sln), while downregulating fast MyHC genes (*Myh2* and *Myh4*; Fig. 3b). Furthermore, Gene Set Enrichment Analysis (GSEA) from whole-genome microarray analysis revealed a strong induction of soleus/slow fibre-specific genes (Fig. 3c). However, Prox1 targets in skeletal muscle were tissue-specific without significant overlap with genes regulated by Prox1 in colorectal cancer^17^,^18^ (Supplementary Fig. 1a).

To test if Prox1 is necessary for the slow fibre program, we deleted Prox1 in skeletal muscle fibres by administering tamoxifen to HSA-Cre^ERT2^;Prox1^fl/fl^ adult mice (indicated as Prox1Δ in figures). We used the human α-skeletal actin (HSA) promoter-driven Cre-recombinase, which is expressed specifically in skeletal muscle fibres^19^ to delete Prox1 in skeletal muscles (Supplementary Fig. 2a). Prox1 deletion resulted in increased expression of fast MyHC genes and downregulation of typical slow fibre genes in soleus muscle (Fig. 3d). As additional evidence, we deleted Prox1 via injection of AAV8-Cre vector into the soleus muscles of Prox1^fl/fl^ mice. This led to a robust decrease of MyHC I protein level in the soleus muscle after three weeks (Fig. 3e, Supplementary Fig. 6a). Collectively, these results showed that Prox1 expression is necessary and sufficient for the slow muscle fibre gene expression program. Furthermore, Prox1-deficient mice had a significantly impaired exercise capacity, as they ran only half of the distance compared with wild-type littermates in a maximal graded exercise test (Supplementary Fig. 2b). In the gastrocnemius muscles of wild-type mice, the expression of Prox1 was decreased after an acute maximal exercise (Supplementary Fig. 2c).

There is a dramatic change in the expression levels of different MyHCs during the first weeks after birth, which was also detected in our experiments (Supplementary Fig. 2d). Surprisingly, however, Prox1 expression in mouse skeletal muscle remained constant during the first three weeks of postnatal life (Supplementary Fig. 2d), suggesting that Prox1 expression is relatively stable during postnatal muscle growth and development.

### Prox1 activates NFAT signalling *in vivo* and *ex vivo*

**Figure 4:**
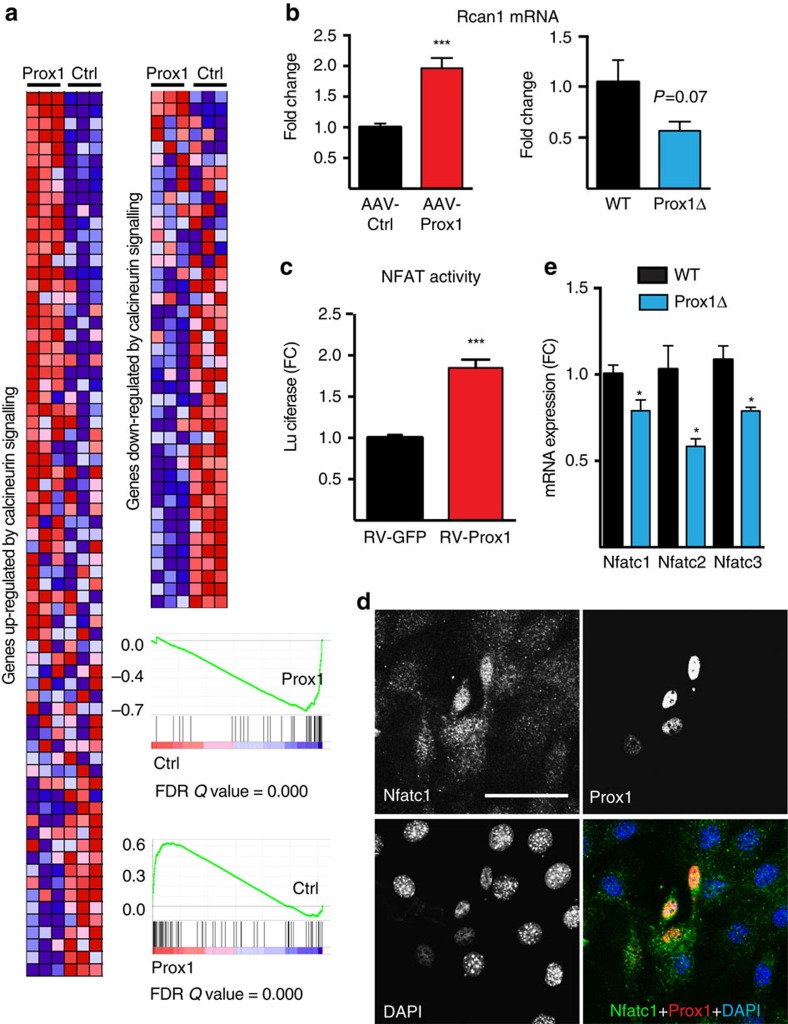
Prox1 acts by activating NFAT signalling. (**a**) Heat maps from GSEA analysis showing that Prox1 overexpression induces the expression of genes known to be upregulated by calcineurin/NFAT signalling (red for upregulation compared with control) and decreases the expression of majority of genes downregulated by calcineurin/NFAT signalling (blue for downregulation compared with control). (**b**) qPCR analysis of regulator of calcineurin-1 (Rcan1) in Prox1 transduced TA muscles and Prox1 deficient soleus muscles. (**c**) NFAT-luciferase reporter assay in C2C12 cells transduced with retroviral (RV) Prox1 or GFP. (**d**) Nfatc1 staining in C2C12 cells overexpressing Prox1. (**e**) Nfatc1–3 mRNA expression in wild-type and Prox1 deleted soleus muscles Data is presented as mean±s.e.m. *n*=3+3 in **a**,**c**, *n*=5+5 and 4+4 in **b**, *n*=3+3 in **d** (repeated three times). Student's two-tailed unpaired *t*-test, *P<0.05, ***P<0.001. Scale bar, 50 μm.

Calcineurin/ Ca^2+^-dependent nuclear factor of activated T cells (NFAT) pathway is a key regulator of slow muscle fibre phenotype^20^. GSEA analysis of the whole-genome transcriptome from Prox1 overexpressing muscles showed a highly significant induction of the genes that are upregulated by NFAT activity, suggesting that Prox1 exerts its effects via this pathway. Consistently, we found that genes repressed by NFAT signalling were downregulated in muscles with Prox1 overexpression (Fig. 4a). In particular, a significant upregulation of Rcan1, which is specifically increased on activation of calcineurin^21^, was detected in the Prox1 overexpressing TA muscles. A decreased Rcan1 expression trend (*P*=0.07, Student's two-tailed *t*-test) in the Prox1 deficient soleus muscles was also observed (Fig. 4b). In addition, Prox1 repressed the expression of key genes inhibiting NFAT activity, such as the muscle-specific calcineurin inhibitor myospryn/*Cmya5* (ref. 22) (log2 FC −0.33, false discovery rate (FDR)=0.007) and calsarcin-2/*Myoz1*^23^ (log2 FC −0.70, FDR=0.03). On the other hand, the expression of α-actinin-2, which competes with calcineurin for binding to calsarcin-2 (ref. 24) and thus functions as a positive regulator of NFAT, was significantly increased (log2 FC 0.66, FDR=0.01). The genes with the most significantly changed expression (FDR<0.001) in AAV-Prox1-treated muscles are listed in Supplementary Dataset.

To test whether Prox1 regulates NFAT activity, we transduced C2C12 myoblasts with a retroviral Prox1 expression vector and subsequently the cells were transfected with an NFAT-luciferase reporter. Prox1-increased luciferase activity in C2C12 cells, indicating that it activates NFAT signalling in myoblasts (Fig. 4c). Immunostaining showed that Prox1 induces nuclear translocation of Nfatc1, reflecting its activation (Fig. 4d). Consistent with this finding, western blotting demonstrated increased cytoplasmic Nfatc1 in Prox1 silenced cells when compared with controls, indicating reduced nuclear translocation of Nfatc1 (Supplementary Figs 3 and 6b). Furthermore, the expression levels of Nfatc1, Nfatc2 and Nfatc3 were downregulated in Prox1 deleted soleus muscles (Fig. 4e). We conclude that Prox1 is involved in the activation of the NFAT pathway and several of its downstream genes in skeletal muscle.

### The Prox1-positive SCs act as stem cells after muscle injury

**Figure 5:**
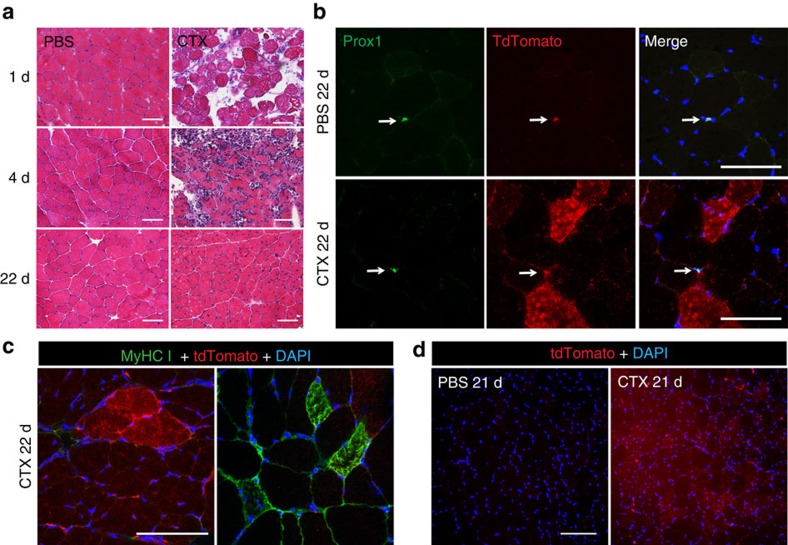
Lineage-tracing experiment of Prox1 expressing satellite cells. (**a**) HE staining of cardiotoxin-treated (CTX) and PBS-injected muscles showing the degeneration and regeneration processes 1, 4 and 22 days after CTX-injection. (**b**) Prox1 staining and TdTomato fluorescence 22 days after CTX-injection. Note several Prox1 negative red fibres, indicating that the Prox1 expressing satellite cells have differentiated into Prox1 negative muscle fibres. No red muscle fibres were found in the PBS-treated muscles. Arrows point to Prox1 and tdTomato-positive SCs. (**c**) The regenerated tdTomato positive muscle fibres originating from Prox1-positive SCs were MyHC I negative in fast tibialis anterior muscle. (**d**) TdTomato-positivity obtained with two tamoxifen injections; one injection was used in the experiments of **a**–**c**. Note that most of the regenerated fibres are red. Scale bars, 100 μm.

Since Prox1 was detected in SC in addition to mature slow muscle fibres, we set out to assess the importance of Prox1 in SC proliferation and differentiation. First, to test the stem cell properties of the Prox1-positive mononuclear cells in skeletal muscle, we used a lineage tracing approach in Prox1-Cre^ERT2^; Rosa26TdTomato^fl/STOP/fl^ mice. In this model, a single tamoxifen injection induces Cre activity and results in the removal of a transcriptional stop cassette in front of the tdTomato reporter coding sequence, thus leading to the expression of the red fluorescent protein exclusively in the Prox1-positive cells and their progenies. Cardiotoxin was injected into the TA muscles to induce muscle injury. As the mouse TA does not contain slow fibres, the Prox1-positive cells in this muscle are SC or lymphatic endothelial cells. After 1 and 4 days, we detected a marked inflammation and degenerating muscle fibres (Fig. 5a). At day 4, the number of TdTomato-positive mononuclear cells was increased in the damaged area, and some of these cells were positive for the proliferation marker Ki67 (Supplementary Fig. 4). At day 22, the cardiotoxin-treated muscles had regenerated and several fibres expressed red fluorescence, demonstrating that they originated from Prox1-positive SCs (Fig. 5b). However, the nuclei of these fibres became Prox1 negative and they did not stain for MyHC I, indicating that they had acquired the fast fibre phenotype typical for TA muscle (Fig. 5c). Muscle fibres with red fluorescence were not found in any of the PBS injected muscles. To increase the labelling efficiency, we injected tamoxifen twice before CTX, which resulted in tdTomato labelling of most of the regenerated fibres three weeks after injury (Fig. 5d). Altogether, these data show that Prox1-positive SCs display stem cell behaviour under regenerative conditions.

### Prox1 is critical for myoblast differentiation

**Figure 6:**
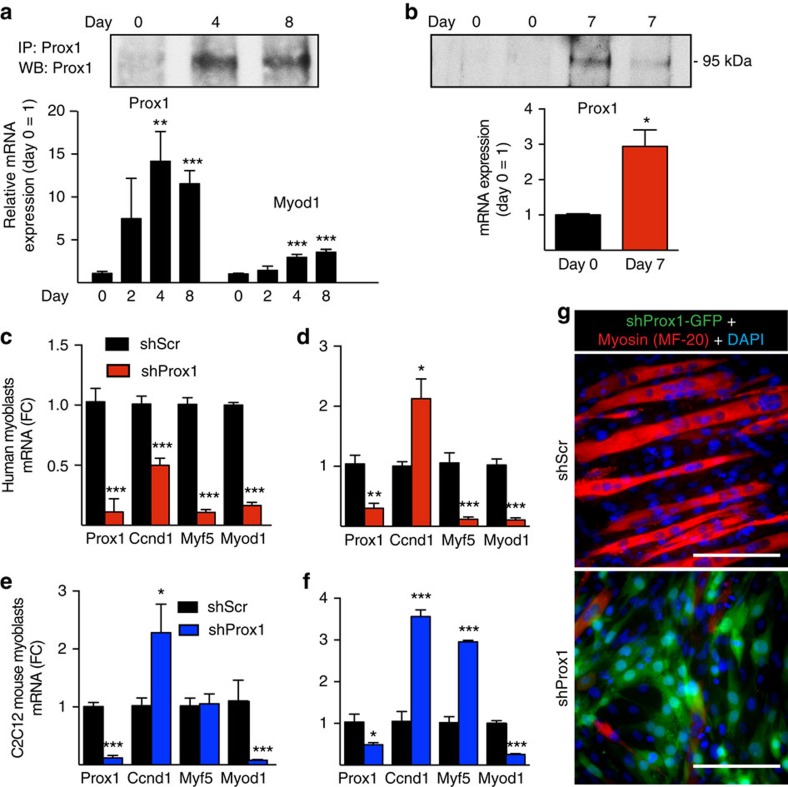
Prox1 regulates myoblast differentiation. (**a**) Analysis of Prox1 mRNA and protein during C2C12 myoblast differentiation (indicated by the increase in MyoD1 mRNA). (**b**) Prox1 protein and mRNA in human myoblasts before and after differentiation. Primary myoblast lines from two different individuals were analysed. (**c**–**f**) Analysis of myoblast proliferation and differentiation-related CyclinD1, Myf5 and MyoD mRNAs in shProx1 and shScr transduced primary human myoblasts before (**c**) and after differentiation for 7d (**d**), as well as in mouse C2C12 myoblasts before (**e**) and after (**f**) differentiation. (**g**) Myosin staining of C2C12 cells after 7d of differentiation. Note that Prox1 silencing completely blocked myotube development, as only occasional cells expressed myosin and these cells were negative for shProx1-GFP. Data is presented as mean±s.e.m., *n*=3+3 biological replicates in **a**–**g** (all experiments were repeated three times). One-way analysis of variance with repeated measures followed by Tukey's posthoc test and Student's two-tailed unpaired *t*-test, *P<0.05, **P<0.01, ***P<0.001. Scale bars, 50 μm.

To further test the role of Prox1 in SCs, we studied the mouse C2C12 myoblast cell line and primary human myoblasts. These cells can differentiate into myotubes with multiple nuclei, thus displaying stem cell features. Prox1 expression was strongly increased during mouse and human myoblast differentiation both at the mRNA and protein level (Fig. 6a,b). Silencing Prox1 by lentiviral shRNA significantly affected the expression of Myod1 and Myf5 in undifferentiated and differentiated human myoblasts (Fig. 6c,d) as well as in C2C12 cells (Fig. 6e,f). This resulted in a dysregulated differentiation of the myoblasts, evidenced by the lack of myosin-positive multinucleated myotubes (Fig. 6g). In addition, we found that CyclinD1 (Ccnd1) mRNA level and the proliferation of Prox1 silenced cells were significantly elevated (EdU positive C2C12 cells after 4 h: shProx1 47.5±1.2 % versus shScr 37.8±0.6 %, *P*<0.001, Student's two-tailed *t*-test), (Fig. 6d–f).

### Bidirectional regulation between Prox1 and Notch1

**Figure 7:**
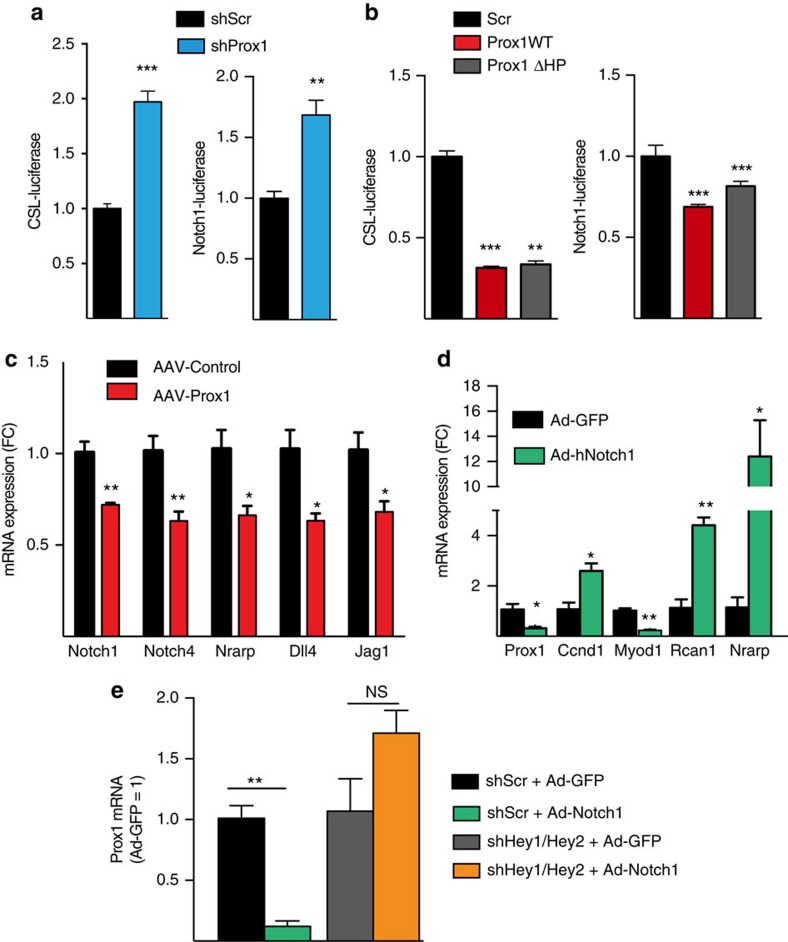
Bidirectional regulation between Prox1 and Notch1 in myoblasts. (**a**) Notch luciferase activity in C2C12 cells transduced with lentiviral shScr or shProx1, and later transfected with CSL- or Notch1-luciferase reporter. (**b**) Effect of overexpression of WT Prox1 and the Prox1 mutant lacking the DNA-binding domain (Prox1 ΔHP) on CSL- and Notch1-luciferase activities. (**c**) Effect of Prox1 overexpression on Notch-related genes *in vivo* in TA muscle. (**d**) Effect of adenoviral Notch1 overexpression on Prox1, CyclinD1, MyoD and Rcan1 mRNAs in human primary myoblasts. Nrarp was used to indicate Notch pathway activation. (**e**) Effect of Hey1 and Hey2 silencing on Notch1-induced suppression of Prox1 expression in human myoblasts. Data is presented as mean±s.e.m., *n*=4+4 biological replicates in **a** and **b** and 3+3 in **d**,**e** (all experiments were repeated two to three times), *n*=5+5 in **c**. Student's two-tailed unpaired *t*-test, *P<0.05, **P<0.01, ***P<0.001.

Notch signalling activity is crucial in maintaining SC quiescence and preventing premature muscle differentiation^25^,^26^,^27^. Since our results showed that Prox1 is needed for the differentiation of myoblasts that have stem cell features, we studied if Prox1 modulates Notch signalling. We first tested if Prox1 regulates Notch signalling activity in C2C12 cells transfected with a CSL-luciferase reporter (for monitoring general Notch activity) or Notch1-specific luciferase reporter. Prox1 silencing significantly increased Notch activity in both assays, indicating that Prox1 represses Notch1 signalling (Fig. 7a). To test the functional domains of Prox1 that are needed for repression of Notch signalling, we transduced myoblasts with lentiviral constructs for wild-type Prox1 or the N-terminal domain of Prox1 lacking the DNA binding domain, and tested their effects on CSL- and Notch-luciferase activities. The results indicated that the N-terminal part of Prox1 was sufficient to suppress Notch1-activity, thus direct interaction with DNA was not required (Fig. 7b). Similar results were also obtained with transient transfection of these constructs (Supplementary Fig. 5a). We then investigated if this regulation occurs *in vivo* in skeletal muscle. Prox1 overexpression in mouse skeletal muscle resulted in significant downregulation of several Notch signalling-related genes (Fig. 7c).

Notch and its downstream effectors Hey1 and Hey2 were shown to regulate Prox1 in lymphatic endothelial cells^28^. Thus, we next tested if Notch activity regulates Prox1 expression in myoblasts. Human primary myoblasts transduced with Ad-hNotch1 exhibited decreased Prox1 expression, whereas inhibition of Notch signalling by administration of the gamma-secretase/Notch cleavage inhibitor DAPT tended to increase Prox1 expression (Fig. 7d and Supplementary Fig. 5b). Notch1 also increased CyclinD1 and decreased MyoD1 expression, which are needed to sustain cells in proliferating and undifferentiated state, respectively. To test if Prox1 expression is regulated by canonical Notch signalling, we silenced both Hey1 and Hey2 in human myoblasts, and found that this abolished the Notch1-induced decrease of Prox1 expression (Fig. 7e). These data suggest that high Notch activity in quiescent and proliferating SCs inhibits Prox1 expression, and that during differentiation, when Notch activity decreases, Prox1 expression is induced. Collectively, these results identify Prox1 as an essential transcription factor required for myoblast differentiation via bidirectional interaction with Notch1.

## Discussion

**Figure 8:**
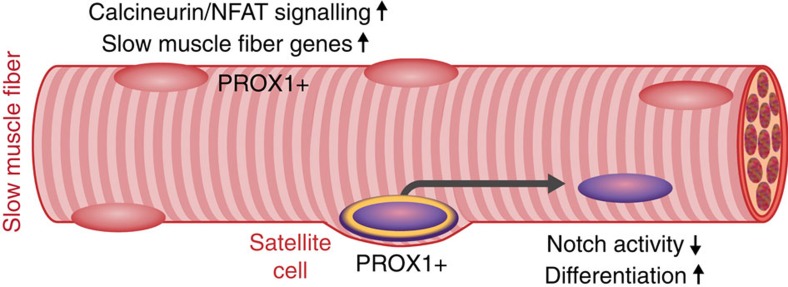
Schematic summary of the pathways regulated by Prox1 in mature skeletal muscle fibres and satellite cells. Prox1 is expressed in mature slow muscle fibres, where it regulates NFAT signalling and muscle fibre type gene programs. Prox1 is also expressed in satellite cells, where it interacts with Notch signalling pathway, and it is essential for myoblast differentiation.

In this study we identified the transcription factor Prox1 as a critical regulator of SC differentiation and muscle fibre phenotype in rodents and humans. Prox1 was necessary and sufficient for the slow fibre type gene program by regulating NFAT signalling and essential for myoblast differentiation by reciprocal regulation, with Notch, (see the schematic in Fig. 8).

Our results show that Prox1 is expressed only in the nuclei of MyHC I positive slow muscle fibres, but not in the fast muscle fibres. In a recent study, Prox1 was shown to suppress fast fibre genes in the heart and skeletal muscle, but the authors suggested that this occurs in all muscle fibre types^16^. We demonstrate here that Prox1 is able to initiate the slow muscle fibre program when transduced into fast muscles and that Prox1 deletion suppresses slow and induces fast fibre genes. Plasticity for fibre type switch can be activated in adult muscles by the previously identified Mef2, NFAT and myogenin transcription factors, which play a role in the determination of fibre-type characteristics^20^,^29^,^30^. The NFAT pathway, especially Nfatc1, has been demonstrated to be essential for slow fibre-type determination^31^,^32^,^33^. Our whole-genome RNA expression analysis of Prox1 overexpressing muscles indicated NFAT pathway activation, thus accounting for the ability of Prox1 to induce slow muscle fibre genes. Furthermore, Prox1 deletion resulted in decreased expression of Nfatc1–3 in skeletal muscle. Prox1 acts mainly as a transcriptional repressor^34^, which is in line with the observed downregulation of the main inhibitors of NFAT signalling in Prox1 overexpressing muscles. These findings were further substantiated by results from the cultured C2C12 myoblasts, where Prox1-induced Nfatc1 nuclear translocation and NFAT transcriptional activation. In line with our results, Prox1 has been shown to be essential for shear stress-induced NFAT activation in lymphatic endothelial cells^35^. Thus, our results provide novel evidence that Prox1 regulates muscle fibre phenotype via NFAT signalling.

In addition to slow muscle fibres, Prox1 is expressed in most, if not all, SCs and its expression is induced during myoblast differentiation. With lineage tracing we obtained evidence that Prox1-positive cells regenerate into muscle fibres after cardiotoxin-induced muscle injury. To reveal the significance of Prox1 in SCs, we silenced Prox1 in human primary myoblasts and in mouse C2C12 myoblasts. This resulted in dysregulated expression of myogenic factors and in complete inhibition of differentiation. Several factors, such as Pax3, Pax7 and MyoD family members, have been shown to be involved in the regulation of SC properties (reviewed in ref. 2). They act in concert at different stages of SC self-renewal and differentiation. Our findings identify Prox1 as a new important regulator of SC differentiation.

Notch signalling is critical for skeletal myogenesis and it has an important role in maintaining the muscle stem cell pool by blocking myogenic differentiation and preventing premature muscle differentiation^25^,^26^,^27^. After muscle injury, SCs start to proliferate and some of them will differentiate into myofibres concomitantly with downregulation of Notch, while others resume a quiescent state^36^. Our results indicate a bidirectional negative regulation between Prox1 and Notch1 in myoblasts and in skeletal muscle, which fits nicely with their sequential expression patterns during the transition from SC quiescence to differentiation. Deletion of the C-terminal DNA binding domain (homeobox and prospero domains) of Prox1 was not sufficient to relieve the Prox1-mediated suppression of Notch1 promoter activity, indicating that Prox1 influences Notch1 as transcriptional co-repressor via (an)other factor(s).

In neuronal progenitor cells, Prox1 has been shown to regulate the Notch1-mediated inhibition of neurogenesis and to directly interact with chromatin at the Notch1 promoter^37^. Similarly to our findings, the DNA binding domain of Prox1 was not needed for the repression of Notch1 activity in neuronal progenitor cells, and the nuclear receptor LHR-1/NR5A2 was identified as a binding partner of Prox1 on the Notch1 promoter. LHR-1/NR5A2 is also expressed in C2C12 cells^38^ and skeletal muscles (unpublished findings of RK and KA), thus it may serve as a binding partner for Prox1 in SC as well. We have recently shown that Prox1 is essential for the expansion of the stem cell pool in intestinal tumours, where its deletion reduced the number of stem cells^17^. Together, our results and previous findings suggest that Prox1 is an important regulator of stem cell behaviour in various tissues.

The present results identify Prox1 as an important factor in skeletal muscle SC differentiation and show that Prox1 is an integral part of the slow muscle fibre program. Our results open up new directions to study and to manipulate SC behaviour during regeneration, and to understand better the role of Prox1 in muscle diseases and aging.

## Methods

### Animal experiments

All mice used in the experiments were maintained in the C57Bl/6J background. Animal experiments were approved by the Regional State Administrative Agency for Southern Finland. Mice were housed in individually ventilated cages with enrichment materials in a facility monitored by the Federation of European Laboratory Animal Science Associations guidelines and recommendations.

The Prox1^fl/fl^ (from Dr G. Oliver) and Human Skeletal Actin-MerCreMer (HSA-Cre^ERT2^, from Dr K. Esser) mice were crossed to generate skeletal muscle fibre specific Prox1-deficient mice (Prox1Δ). These mouse lines have been described previously^19^,^39^. To delete Prox1 from skeletal muscles, 8–10 weeks old male mice were given tamoxifen i.p. (80 mg/kg) for five consecutive days. The muscles were collected and analysed after two months of deletion. The experiment was performed twice with independent cohorts (*n*=4+4 and *n*=6+8). To delete Prox1 only in soleus muscle, AAV9-Cre or empty vector (30 μl per muscle, 6.96 × 10^9^ vp ml^−1^) were injected into soleus muscles of Prox1^fl/fl^ mice (*n*=3+4 and in a second experiment *n*= 5+6).

For overexpression of Prox1 in fast skeletal muscle, 8 weeks old C57Bl/6J male mice (C57Bl/6JOlaHsd from Harlan) were anaesthetized and recombinant adeno-associated viral vector (AAV8) encoding Prox1 was injected into mouse TA muscle (30 μl per muscle, 6.06 × 10^9^ vp ml^−1^). The contralateral muscle was injected with AAV8 encoding mCherry reporter protein equipped with a nuclear localization signal. The experiment was performed twice with independent cohorts (*n*=3+3 and *n*=5+5).

Whole calf muscle complex was collected from wild-type mice at postnatal days 0, 7 and 21 to study the expression of Prox1 during postnatal development (*n*=3 for each time point).

To investigate the role of Prox1 in SC, we performed lineage-tracing experiments. Prox1-Cre^ERT2^ mice^40^ (from Dr G. Oliver) were crossed with Rosa26-tdTomato^fl/Stop/fl^ (Jackson Laboratories, Stock 007914) reporter mice. To label a subset of Prox1 positive cells, mice were given one tamoxifen dose (3 mg) at the same time when cardiotoxin (50 μl of 10 μM) was injected into TA muscle. PBS was injected into the contralateral muscle. Muscles were collected and analysed 1, 4 and 22 days after the injection (*n*=4 muscles/time point/treatment). In another experiment, we increased the tamoxifen dose to get more efficient labelling and gave two injections one and two days before cardiotoxin administration.

Maximal endurance running exercise test was performed on a rodent treadmill (Panlab) according to the following protocol: 9 m min^−1^ for 5 min, 12 m min^−1^ for 5 min, 15 m min^−1^ for 5 min, 18 m min^−1^ for 5 min and 20 m min^−1^ until exhaustion (10° incline). Adaption training session was performed two times before the experiment.

In all experiments using wild-type mice, mice were randomized into the experimental groups based on their body weight. The investigators were blinded to group allocation during experiments.

Soleus, gastrocnemius and tibialis anterior muscles were collected from three months old male wild-type rats for RNA and immunohistochemical analyses (*n*=4).

### Cell culture

The mouse myoblast cell line C2C12 (ATCC) was cultured in growth medium (DMEM high glucose) containing 10% fetal bovine serum (FBS), 2 mM L-glutamine, penicillin (100 U ml^−1^) and streptomycin (100 U ml^−1^). Cells were differentiated for 4, 6, 7 and 8 days for further experiments. Differentiation medium contained (DMEM high glucose), 1–2% horse serum (HS), 2 mM L-glutamine, penicillin (100 U ml^−1^) and streptomycin (100 U ml^−1^).

The isolation of SC and preparation of primary human myotubes were conducted as described previously^41^. In brief, a muscle biopsy of about 100 mg was taken under local anaesthesia with lidocaine (10 mg ml^−1^, without epinephrine) from the *vastus lateralis* muscle of healthy men. The tissue was dissected into small pieces followed by trypsinization in a shaking water bath at 37 °C. SC were then isolated and maintained in DMEM-F12 culture medium containing 20% FBS, 1% penicillin, 1% streptomycin, and 1% fungizone. Primary myoblasts were selected via magnetic cell separation using the CD56 antibody (Miltenyi Biotec). To obtain myotubes, myoblasts of 80% confluence were switched to differentiation medium containing 2% FBS for 7 days. The human study was approved by the Ethical Committee of Department of Medicine, Helsinki University Central Hospital and written informed consent was obtained from all participants.

Lentiviral Prox1 silencing construct (shProx1) and scramble control (shScr) were from the TRC1 library for human myoblasts (Sigma-Aldrich)^17^. For mouse myoblasts we used shProx1 constructs from TRC1 and TRC2 (TRCN0000070723, TRCN0000070724, TRCN0000420735, TRNC0000425447, TRCN0000432522 from Sigma-Aldrich). The lentivirus-containing supernatant of 293FT cells was concentrated, and cells were transduced overnight with polybrene (Sigma, 8 μg ml^−1^). After transduction, cells were further selected with 3 μg ml^−1^ (human myoblasts) or 30 μg ml^−1^ (C2C12) puromycin (Sigma). After selection, the cells were cultured for further experiments. Retroviral constructs for Prox1 and GFP (control) were used for transfection of undifferentiated C2C12 cells overnight, followed by recovery for two to three days before the experiments.

Prox1-overexpressing and -silenced cells were transfected with expression plasmids encoding the NFAT-luciferase reporter (NFAT/AP-1 3 × luciferase was a gift from Anjana Rao, Addgene plasmid #11783), Notch reporter CSL-luciferase reporter (4xCSL-luciferase was a gift from Raphael Kopan, Addgene plasmid # 41726) or Notch1-luciferase reporter^37^,^42^ (gift from Tohru Kiyono) by using the jetPEI reagent (Polyplus transfection). TK-Renilla luciferase was used as a transfection control. Promega's dual luciferase assays were performed according to the manufacturer's instructions. All experiments were done in quadruplicate and repeated at least 2–4 times.

To analyse the effect of Notch signalling activity on Prox1 expression, we transduced primary human myoblasts with adenoviral constructs encoding human Notch1 or GFP as a control. To inhibit Notch activity, the cells were treated with gamma-secretase inhibitor DAPT (10 μM). Prox1 expression was analysed after 2–3 days of treatment. To silence Hey1 and Hey2 in human myoblasts, lentiviral vectors were produced from TRC1 library constructs (TRCN00000202014 and TRC0000020249, Sigma-Aldrich).

For generation of the PROX1 mutant without DNA binding domain (PROX1-deltaHP), the stop codon was introduced after nucleotides encoding Ser569 (XP_005273251.1|) in the construct encoding a MYC-tagged wild type human PROX1 in the lentiviral vector pSD44 (ref. 43) using standard site-directed mutagenesis procedures. The presence of mutation was confirmed by sequencing, and protein truncation and equivalent wild type and mutant protein production levels were verified by western blot analysis.

### RNA extraction and quantitative real-time PCR

Cells were lysed and total RNA was extracted with Nucleospin RNA II Kit (Macherey-Nagel). Muscle tissues were first lysed with Trisure reagent (Bioline) and RNA was further extracted with the Nucleospin RNA II Kit (Macherey-Nagel). cDNA synthesis was performed with the iScript cDNA synthesis kit (Bio-Rad) using 1 μg RNA and random hexamer primers. qRT-PCR was performed with the iQ SYBR green supermix (Bio-Rad). Relative gene expression levels were calculated using the formula 2^−ΔΔCt^. Primers are listed in Supplementary Table 1.

### Whole-genome microarray

The quality of RNA was determined with Bioanalyzer (Agilent Technologies) and analysed on genome-wide Illumina Mouse WG-6 v2 Expression BeadChips (Illumina). Illumina's GenomeStudio software was used for initial data analysis and quality control and the further data analysis was performed with the Chipster software (www.chipster.csc.fi)^44^. After quantile normalization, statistically significant differences in individual genes between the groups were tested using Empirical Bayes statistics and the Benjamini-Hochberg algorithm controlling FDR. Adjusted FDR values of *P*<0.05 were considered significant. The microarray data have been submitted to the GEO database, under the series accession number GSE69199.

### Gene set enrichment analysis

The gene expression dataset was transferred to the Gene Set Enrichment Analysis software (http://www.broadinstitute.org/gsea)^45^,^46^ and the analysis was carried out with default parameters except that the ‘exclude smaller sets' was set to 25 and gene permutation was applied. We used a modified list of the kegg.v3.1.symbols gene set (http://www.broadinstitute.org/gsea/msigdb), with the addition of slow muscle fibre-specific genes^47^ and Prox1 target genes in colorectal cancer^18^. FDR *Q* value <0.1 was regarded significant.

### Polyacrylamide gel electrophoresis and western blotting

Cells were lysed with cold RIPA buffer (50 mM Tris-HCl at pH7.6, 150 mM NaCl, 1% NP-40, 0.5% DOC, 0.1% SDS, with protease and phosphatase inhibitors). Muscle tissues were lysed with the same buffer using ceramic beads. The concentration of the lysates was measured with the BCA protein assay kit (Thermo Scientific) following the manufacturer's instructions. The samples were loaded to SDS-polyacrylamide gel (Bio-Rad Laboratories), proteins were transferred to nitrocellulose membranes and incubated with primary antibody against MyHC I (BA-D5, Developmental Studies Hybridoma Bank, 1:250), Nfatc1 (YT5381, Immunoway, 1:1,000) or HSC70 (Santa Cruz Biotechnology, 1:5,000). For Prox1, cell lysates and soleus muscle homogenates were first immunoprecipitated with rabbit-anti-human Prox1 (in-house made, 1:1,000) antibody using Protein G beads, transferred to nitrocellulose membranes and incubated with primary antibody for Prox1 (goat-anti-human, AF2727, R&D, 1:1,000). HRP-conjugated anti-mouse or anti-goat secondary antibodies (Dako, 1:5,000) were used and the signal was visualized by the SuperSignal West Pico or Femto Maximum Sensitivity Substrate (Thermo Scientific). Alternatively, anti-rabbit IRDye800 and anti-mouse IRDye680 were used for detection (Licor, 1:10,000).

### Histochemistry and immunofluorescence

Mouse, rat (soleus, gastrocnemius and tibialis anterior) and human (vastus lateralis) muscle samples were frozen in liquid nitrogen-cooled isopentane in Tissue-Tek OCT. 10 μm sections were stained with hematoxylin-eosin staining and using the following primary antibodies: PROX1 (AF2727, R&D, 1:500 dilution), Laminin (Thermo Scientific, 1:500), CD31 (BD Biosciences, 1:500), MyHC I (BA-D5, Developmental Studies Hybridoma Bank, 1:50), Pax3 and Pax7 (Developmental Studies Hybridoma Bank, 1:50) and NCAM/CD56 (559043, BD Biosciences, 1:200), red fluorescence protein (600-901-379, Rockland Immunochemicals, 1:500) and Ki67 (Leica, 1:200). For signal detection, Alexa Fluor 488, 594, 633- or 647-conjugated secondary antibodies (Molecular Probes, 1:500) were used. Nuclei were labelled with DAPI. C2C12 cells and primary human myoblasts were fixed with 4% paraformaldehyde for 10–15 min and stained with phalloidin (F-actin, Molecular Probes, 1:500) for cell morphology and desmin antibody (Millipore, 1:500) to confirm that the human primary cells were of muscle origin. C2C12 cells were also stained with Nfatc1 (YT5381, Immunoway, 1:100) and myosin (MF-20, Developmental Studies Hybridoma Bank, 1:50) antibodies. For proliferation studies, shScr and shProx1 C2C12 cells were incubated for 4 h on coverslips with EdU and stained according to the manufacturer's protocol (Molecular Probes). Samples were imaged using Leica DM LB light microscope, Zeiss Axioplan fluorescent microscope and Zeiss LSM 780 confocal microscope. Images were processed and analysed with ImageJ (NIH).

### Statistical analysis

Statistical comparison of two groups was done by Student's two-tailed unpaired *t*-test or one-way analysis of variance (with repeated measurements in C2C12 cell differentiation experiment) followed by Tukey's *post hoc* test for more groups using the Prism 6.0 software. Data was checked for normality and equal variances between groups. *P*<0.05 was considered as statistically significant and the significance is marked by *P<0.05, **P<0.01, and ***P<0.001. The required sample size was calculated based on the similar experiments and analyses carried out previously. The number of animals in each experiment is stated in the respective figure legends. All *in vitro* experiments were replicated 2–4 times and the main animal experiments were conducted twice or using different species. Samples or animals were excluded from the data analysis with pre-established criteria, if they deviated more than 2 s.d. from the group mean.

### Data availability

The microarray data have been submitted to the GEO database, under the series accession number GSE69199.

## Additional information

**How to cite this article:** Kivelä, R. *et al*. The transcription factor Prox1 is essential for satellite cell differentiation and muscle fibre type regulation. *Nat. Commun.* **7,** 13124 doi: 10.1038/ncomms13124 (2016).

## Supplementary Material

Supplementary Information Supplementary Figures 1-6 and Supplementary Table 1

Supplementary Dataset 1 The most significantly changed genes in mouse TA muscle transduced with AAV8-Prox1 (FDR<0.001).
