# Supplementary material for: The transcription factor Prox1 is essential for satellite cell differentiation and muscle fibre-type regulation
Source: Nat Commun. 2016 Oct 12;7:13124. doi: 10.1038/ncomms13124 (PMC5064023; doi:10.1038/ncomms13124)
Supplement: Supplementary Information — Supplementary Figures 1-6 and Supplementary Table 1 [file ncomms13124-s1.pdf]

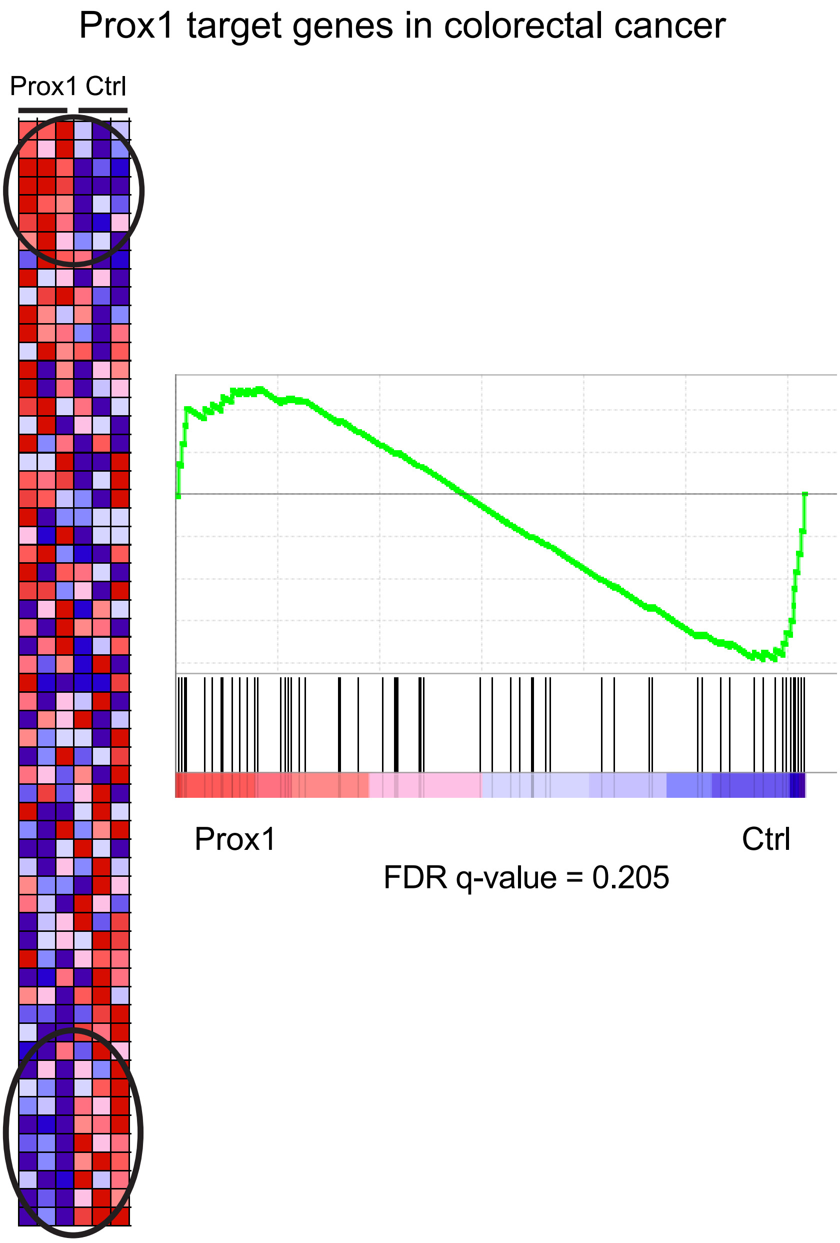

Supplementary Figure 1. Most of the Prox1 target genes in colorectal cancer cells are not Prox1 regulated in skeletal muscle. Heat map from GSEA analysis, showing that Prox1 overexpression induces changes in only a small subset of genes shown to be affected in colorectal cancer cells (red: up-regulation and blue: down-regulation compared to control), indicating tissue specificity of Prox1 regulation. (n = 3+3)

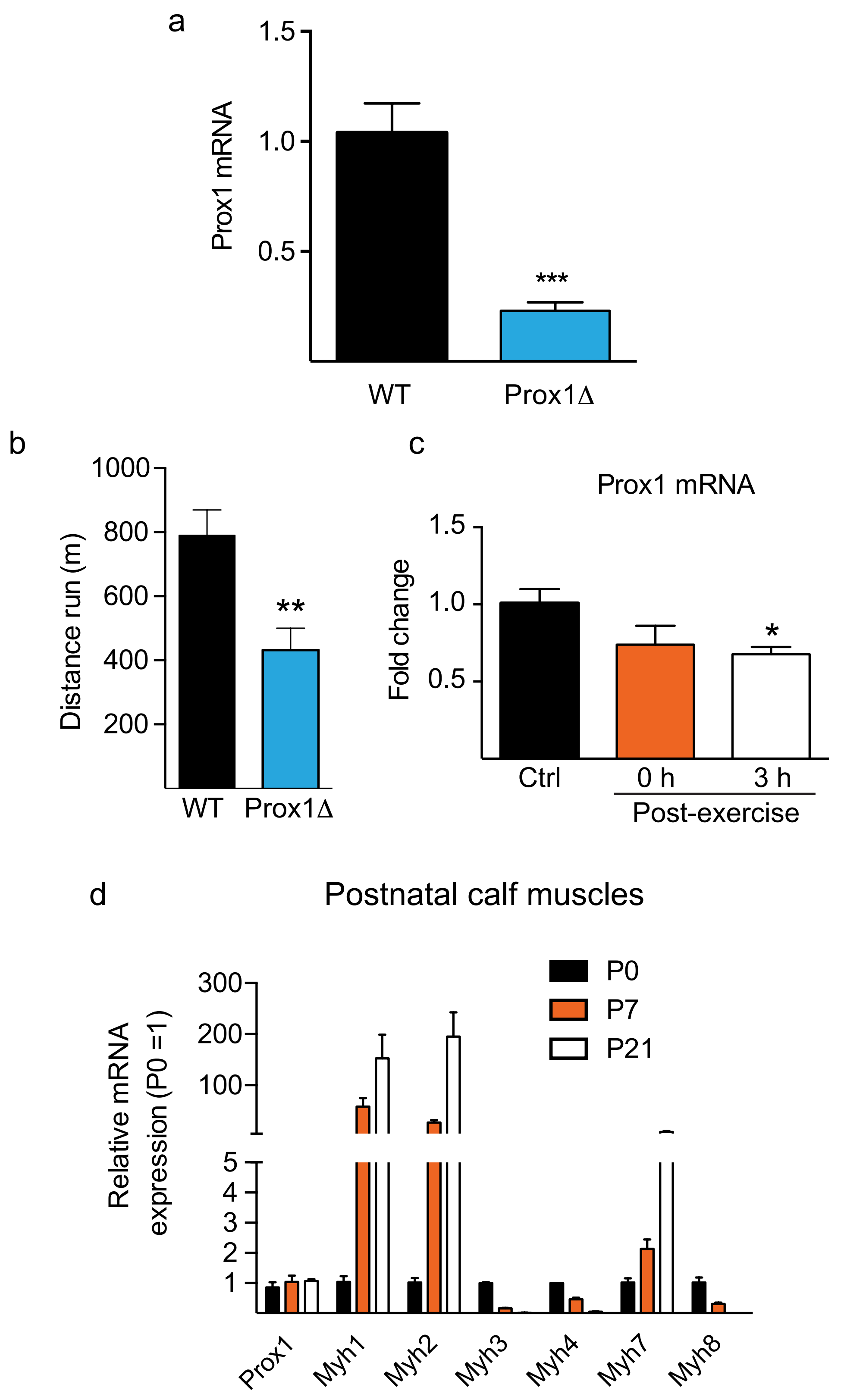

Supplementary Figure 2. Maximal exercise capacity in Prox1Δ mice and the expression of Prox1 and myosin heavy chain genes during postnatal skeletal muscle development. (a) Prox1 expression in HSA-MCM;Prox1fl/fl (Prox1Δ) and in Prox1fl/fl (WT) soleus muscles. (b) Maximal exercise capacity of WT and Prox1Δ mice in a maximal treadmill running test. (c) Prox1 expression in gastrocnemius muscles of WT mice after the acute maximal running exercise. (d) RNA expression in the calf muscles during P0-P21. Note that Prox1 RNA remains stable, whereas the fast and slow myosin heavy chain mRNAs increase and embryonic/neonatal Myh3 and Myh8 decrease markedly during postnatal development. Data is presented as mean ± s.e.m, n = 6+8 in (a), n = 5+5 in (b), n = 3+4+4 in (c) and n=3 for each time point in (d), Student's two-tailed unpaired t-test and One-way ANOVA with Tukey's post hoc test, *$p<0.05$, **$p<0.01$, ***$p<0.001$

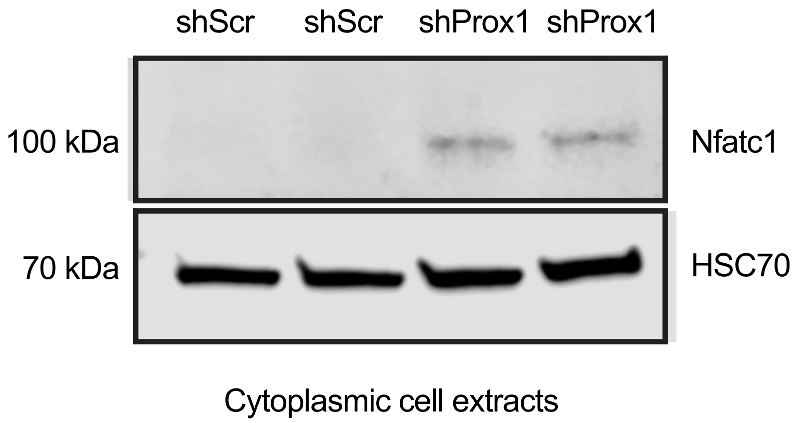

Supplementary Figure 3. Prox1 affects nuclear localisation of Nfatc1. Nfatc1 protein was analysed in cytoplasmic lysates from shScr and shProx1 C2C12 myoblasts. Triton-X100 and NP-40 (which do not solubilize the nuclei) were used as detergents in the lysis buffer. HSC70 was used as a loading control in gel electrophoresis.

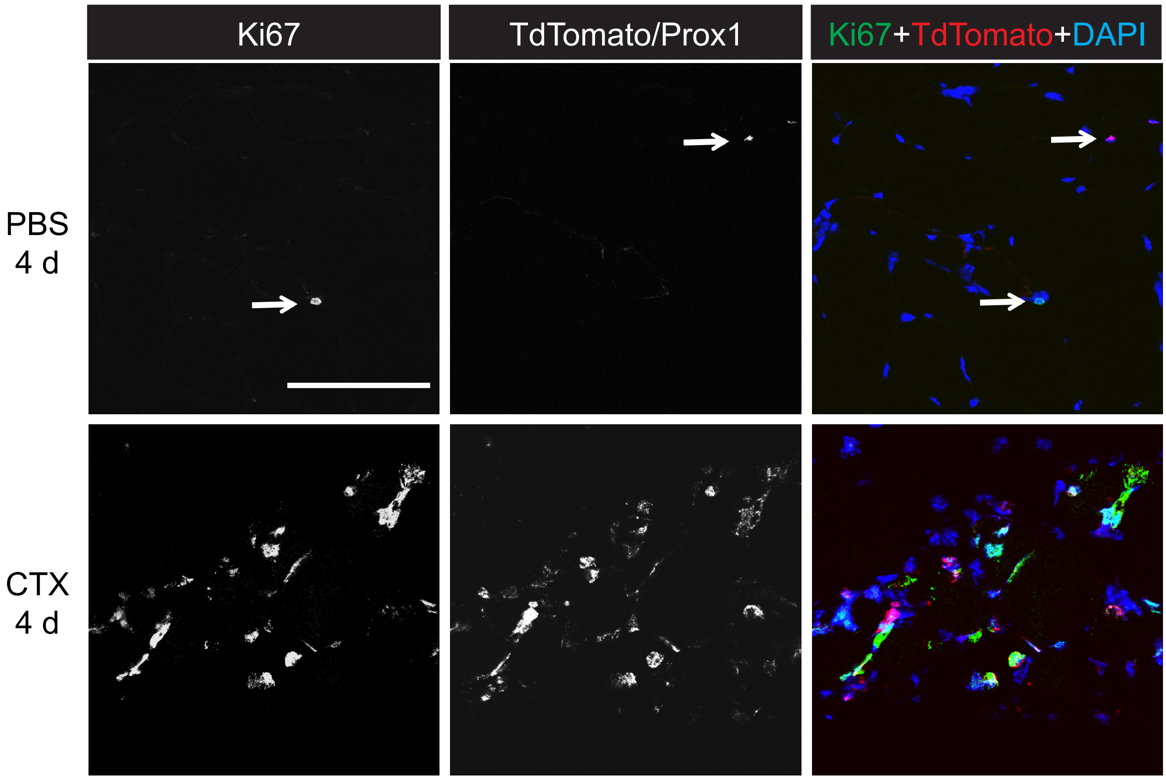

Supplementary Figure 4. Proliferation of Prox1-positive satellite cells after muscle injury. Staining of the Ki67 cell cycle marker and TdTomato Prox1 lineage marker four days after muscle injury. Note that several of the TdTomato-positive cells are also Ki67-positive. Scale bar 100 µm.

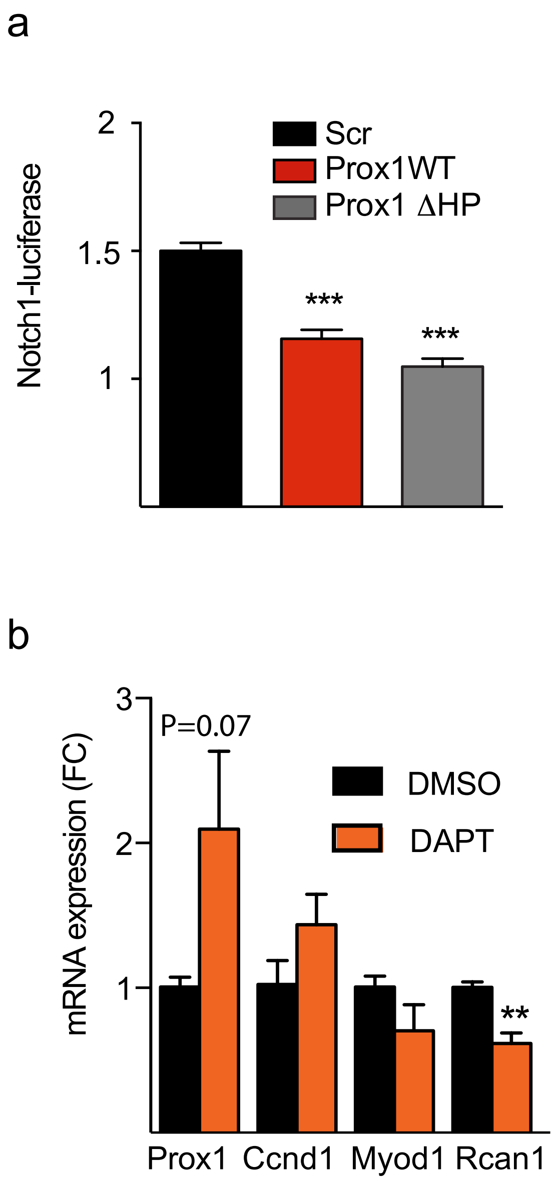

Supplementary Figure 5. Bi-directional signalling between Notch1 and Prox1. (a) Notch-1 luciferase activity in C2C12 myoblasts transiently transfected with Scrambled control vector, WT Prox1 or mutant Prox1 lacking the DNA-binding domain. (b) Effect of DAPT Notch-signalling inhibitor on the mRNA expression of Prox1 and differentiation-related genes. The C1C12 cells were subjected to DAPT for 48 h. Data is presented as mean ± s.e.m, n = 3+3, Student's two-tailed unpaired t-test and One-way ANOVA with Tukey's post hoc test, $^{**}p<0.01$, $^{***}p<0.001$.

a Related to Fig. 3e

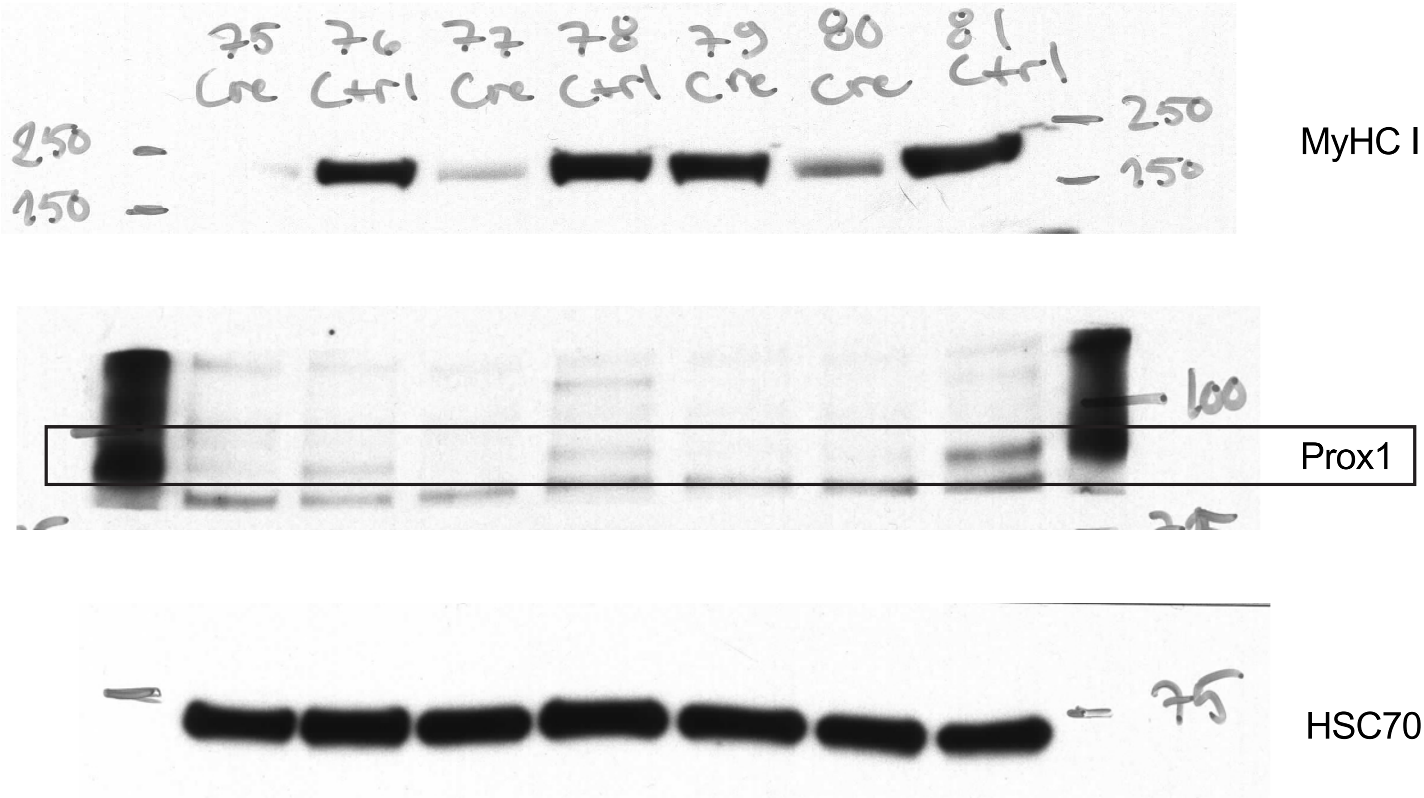

b Related to Supplementary Fig. 3

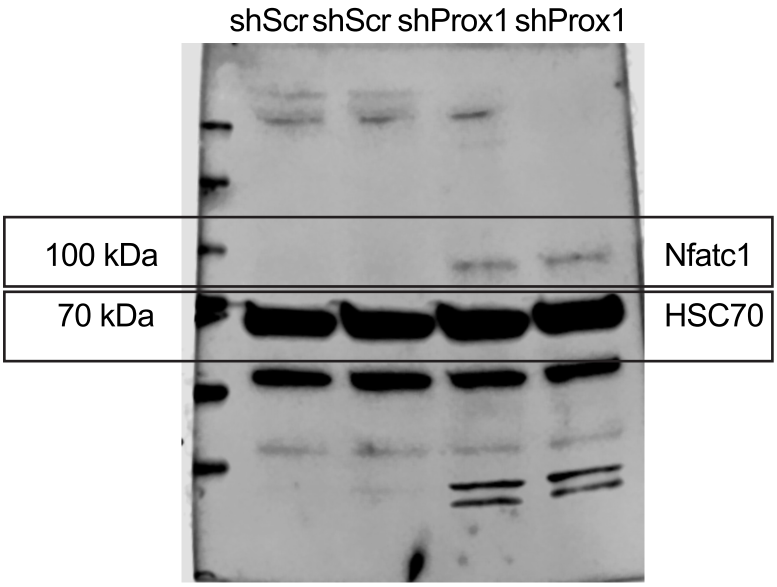

Supplementary Figure 6. Uncropped images of immunoblots with molecular weight markers. The immunoblots presented here are the uncropped images for their corresponding figures in main manuscript. Bands marked in the rectangles were used when there are multiple bands in the same immunoblots.

Supplementary Table 1. Primer sequences.

	Forward	Reverse
hPROX1	5'-TCACCTTATTCGGGAAGTGC-3'	5'-GTACTGGTGACCCCATCGTT-3'
hMYOD1	5'-AATAAGAGTTGCTTTGCCAG-3'	5'-GTACAAATTCCCTGTAGCAC-3'
hMYF5	5'-AAGAGCAGTACTTTTGACAG-3'	5'-GTCCACTATGTTGGATAAGC-3'
hCCND1	5'-GCGGAGGAGAACAAACAGAT-3'	5'-TGAGGCGGTAGTAGGACAGG-3'
hRCAN1	5'-AGACCTTACACATAGGAAGC-3'	5'-GAGATCATAGTTTATGACTGGG-3'
hHEY1	5'-CCGGATCAATAACAGTTTGTC-3'	5'-CTTTTTCTAGCTTAGCAGATCC-3'
hHEY2	5'-TCAAAAGCAGTTGGCACAAG-3'	5'-GATTGAATTCTCCAACAACAACA-3'
hHPRT1	5'-TGAGGATTTGGAAAGGGTGT-3'	5'-TCCCCTGTTGACTGGTCATT-3'
rProx1	5'-GGGACACAACGAGTCTGAGGACCA	5'-GCATGTTGGAGCTGGGGTAACGG-3'
rHprt1	5'-ATGGACTGATTATGGACAGGACTG-	5'-TCCAGCAGGTCAGCAAAGAAC-3'
mProx1	5'-GCTACCCCAGCTCCAACATGCT-3'	5'-TGATGGCTTGACGCGCATACTTCT-3'
mGAPDH	5'-ACAACTTTGGCATTGTGGAA-3'	5'-GATGCAGGGATGATGTTCTG-3'
mTBP	5'-GAAGCTGCGGTACAATTCCAG-3'	5'-CCCCTTGTACCCTTCACCAAT-3'
mMyh1	5'-CTCTTCCCGCTTTGGTAAGTT-3'	5'-CAGGAGCATTTCGATTAGATCCG-3'
mMyh2	5’-GGCACAAACTGCTGAAGCAGAGGC	5'-GGTGCTCCTGAGGTTGGTCATCAGC-3'
mMyh3	5'-CCAAAACCTACTGCTTTGTGGT-3'	5'-GGGTGGGTTCATGGCATACA-3'
mMyh4	5'-TTCATCAGAATCCATTTCGG-3'	5'-TCAGAAGCATCTCAATAAGC-3'
mMyh7	5’-GCCAACTATGCTGGAGCTGATGCCC	5’-GGTGCGTGGAGCGCAAGTTTGTCATAAG-3'
mMyh8	5'-CTGTACGACCAACATCTGGGA-3'	5'-GCACTAGCGTATGTGGAAAAGA-3'
mTnnC1	5'-GCCACAGGTGAGACCATTACG-3'	5'-ATTCGGCCATCGTTGTTCTTG-3'
mTnnI1	5'-GAAAGAAGACACAGAAAAGGAG-3'	5'-AGCAGAAAGATAGGTGAGTG-3'
mMyoz2	5'-ATTGGGATTCACCCCTGCTG-3'	5'-ATCTTAAACAGCCTGGCCCC-3'
mSln	5'-GGTCCTTGGTAGCCTGAGTG-3'	5'-CGGTGATGAGGACAACTGTG-3'
mMyod1	5'-CGGCTACCCAAGGTGGAGAT-3'	5'-ACCTTCGATGTAGCGGATGG-3'
mMyf5	5'-TATGAAGGCTCCTGTATCCC-3'	5'-ACGTGCTCCTCATCGTCTG-3'
mCcnd1	5'-TCCTCTCCAAAATGCCAGAG-3'	5'-GCAGGAGAGGAAGTTGTTGG-3'
mRcan1	5'-CTCAGACTTTACACATAGGAAG-3'	5'-GCATGCAGTTCATACTTCTC-3'
mNFATC1	5'-CCAAGTCAGTTTCTATGTCTG-3'	5'-ATAATTGGAACATTGGCAGG-3'
mNFATC2	5'-TGGATGACGAGTTGATAGAC-3'	5'-GATTTCTCGGATCAAAGACC-3'
mNFATC3	5'-CTGAACCTGAAGATCAAGAAC-3'	5'-TCTCGTTCACATCATCTAAGG-3'
mNFATC4	5'-GTTTCAAGTTCCTACCTGTG-3'	5'-CCTTCTGACAGATAAGGAGTC-3'
mNotch1	TaqMan Mm00435245_m1	
mNotch4	TaqMan Mm00440525_m1	
mNrarp	TaqMan Mm00482529_s1	
mDll4	TaqMan Mm00444619_m1	
mJag1	TaqMan Mm00496902_m1	
